# Probiotic supplements prevented oxonic acid-induced hyperuricemia and renal damage

**DOI:** 10.1371/journal.pone.0202901

**Published:** 2018-08-24

**Authors:** Fernando E. García-Arroyo, Guillermo Gonzaga, Itzel Muñoz-Jiménez, Mónica G. Blas-Marron, Octaviano Silverio, Edilia Tapia, Virgilia Soto, Natarajan Ranganathan, Pari Ranganathan, Usha Vyas, Anthony Irvin, Diana Ir, Charles E. Robertson, Daniel N. Frank, Richard J. Johnson, L. Gabriela Sánchez-Lozada

**Affiliations:** 1 Laboratory of Renal Physiopathology, INC Ignacio Chavez, Mexico City, Mexico; 2 Dept.of Pathology, INC Ignacio Chavez, Mexico City, Mexico; 3 Kibow Biotech, Newtown Square, PA, United States of America; 4 Division of Infectious Diseases, University of Colorado, Aurora, CO, United States of America; 5 Renal Diseases and Hypertension, University of Colorado, Aurora, CO, United States of America; University Medical Center Utrecht, NETHERLANDS

## Abstract

Hyperuricemia is highly prevalent and especially common in subjects with metabolic, cardiovascular and renal diseases. In chronic kidney disease, hyperuricemia is extremely common, and uric acid (UA) excretion relies on gut uricolysis by gut microbiota. Current therapy for lowering serum UA includes drugs that may produce undesired secondary effects. Therefore, this pilot study was designed to evaluate the potential of two probiotic supplements to reduce systemic uric acid concentrations. Secondary objectives were to assess whether the hypouricemic effect related to a therapeutic benefit on the hyperuricemia-induced renal damage and hypertension. Analysis of fecal microbiota was also performed. Groups of 6 rats each were followed for 5 weeks and allocated in the following treatment groups: C = Control; HU-ND = Oxonic acid-induced hyperuricemia (HU) +regular diet; HU-P = HU+placebo; HU-F1 = HU+ probiotics formula 1 and HU-F2 = HU+ probiotics formula 2. We confirmed that oxonic acid-induced hyperuricemia produced hypertension and renal functional and structural changes, along with modest changes in the overall composition of fecal microbiota. Both probiotic-containing diets prevented HU, elevated UA urinary excretion and intrarenal UA accumulation induced by oxonic acid. The hypouricemic effect conferred by probiotic supplementation also prevented the renal changes and hypertension caused by hyperuricemia. However, probiotic treatment did not restore the fecal microbiota. In conclusion, we demonstrated for the first time the ability of probiotics containing uricolytic bacteria to lower serum uric acid in hyperuricemic animals with beneficial consequences on blood pressure and renal disease. As probiotics supplements are innocuous for human health, we recommend clinical studies to test if probiotic supplements could benefit hyperuricemic individuals.

## Introduction

Hyperuricemia is often present in the population, with some studies reporting a prevalence as high as 21% in men and women [1]. Hyperuricemia is especially common in subjects with metabolic, cardiovascular and renal diseases [2–4]. While the presence of hyperuricemia in the absence of gout has often been described as “asymptomatic”, recent studies suggest hyperuricemia may have a contributory role in metabolic and cardiovascular diseases. Potential mechanisms involved in the deleterious metabolic effects of uric acid (UA) include the ability of soluble uric acid to increase oxidative stress, mitochondrial and endoplasmic reticulum dysfunction, endothelial dysfunction, activation of the renin-angiotensin system, as well as increased synthesis and secretion of proinflammatory factors [2–4]. Depending on the cell type, UA may also cause increased proliferation (VSMC) [5, 6] or apoptosis (renal tubular cells, endothelial cells) [7, 8]. While UA is a prooxidant in the intracellular environment, uric acid may also function as an anti-oxidant and has been proposed to protect the central nervous system in conditions such as Parkinson’s disease [9]

Extrarenal excretion of uric acid via the intestine accounts for up to one-third of its total excretion [10]. Uric acid is secreted into the gut where it is rapidly metabolized by bacterial microbiota [10]. Moreover, it was recently shown that gouty patients have a significantly different intestinal microbiota in comparison to normouricemic subjects [11], a finding that suggests an interaction between the microbiota and intestinal UA metabolism and excretion that could potentially modulate serum uric acid levels. In addition, transporter-mediated intestinal secretion or uptake of UA is a significant step in its metabolism by gut microbiota [12, 13]. Endogenous uric acid is secreted from blood directly into the intestinal lumen at all intestinal segments [12]. In humans, some polymorphic variants of the transporter BCRP (also known as ATP-binding cassette transporter, sub-family G, member 2, ABCG2), which secretes UA into the intestine, result in decreased transporter activity, elevated serum uric acid (SUA) and increased incidence of gout [14]. Moreover, blockade of UA transport into the intestine induced an increment in serum UA concentrations, again suggesting that intestinal mechanisms are essential to modulation of UA systemic levels [12]. Another transporter, SLC2A9, is also important in intestinal secretion, and knockdown of intestinal SLC2A9 can also result in hyperuricemia and features of metabolic syndrome [15].

Probably the population that most depends on gut excretion and metabolism are those subjects with end-stage renal disease who have not yet been started on dialysis and in which hyperuricemia is extremely common [16, 17] [18]. In these patients, UA excretion relies on intestinal uricolysis by gut microbiota. Hyperuricemia also has been associated with the risk of developing acute kidney injury [19–21] and with the progression of chronic kidney disease [22–25]. Thus, in these pathologies, the use of drugs aimed to reduce SUA has accomplished promising results [26–29].

Current therapy for lowering serum UA includes inhibitors of xanthine oxidase (allopurinol, febuxostat), recombinant uricase (rasburicase) and uricosuric agents (probenecid). Nevertheless, all these drugs may produce undesired secondary effects. [30]. Therefore, the development of alternative therapeutic strategies to reduce UA concentrations would be useful. Thus, this pilot study was designed to evaluate the potential of two probiotic supplements to lower systemic uric acid concentrations. Secondary objectives were to evaluate whether hypouricemia was accompanied by a therapeutic benefit on the hyperuricemia-induced renal damage and hypertension. Finally, we profiled fecal microbiota in order to assess the effects of hyperuricemia and probiotic supplementation on bacterial community structure.

## Methods

### Experimental animals

All animal procedures were conducted in strict accordance with the Guidelines for Care and Use of Laboratory Animals and were approved by the Animal Care and Use Committee at the Instituto Nacional de Cardiologia Ignacio Chávez (Protocol number INC/CICUAL/007/2016). All efforts were made to minimize animals suffering.

Thirty male Wistar rats were ordered from Envigo Mexico (Mexico City, Mexico), and given 5 days to acclimate to the housing facility prior and be trained for baseline systolic blood pressure (SBP) measurement. Rats were housed in micro-barrier system cages 491 X 273 X 272 mm (Allentown Inc MBS101968HT. NJ, USA) and given access to food and water ad libitum during acclimation. Environmental enrichment included bedding (GreenSoft, RGS-Mexico. Mexico City and a black acrylic tube (l = 175 mm, OD = 70 mm, ID = 60 mm). Animals were monitored on a daily basis for health status. Baseline urine and fecal samples were collected by placing rats in metabolic cages (Tecniplast. Varese, Italy) for 16 h with food and water ad libitum. SBP was measured by tail-cuff manometry in conscious animals previously accustomed to this procedure (NIBP System IN125/R. ADInstruments Inc. Dunedin, New Zealand). Three consecutive measurements were recorded, and the mean reported. After SBP quantification a sample of blood was taken from the tail vein (600 μL), and plasma stored at -20°C until further processing.

### Experimental protocol

Five groups of 6 rats each were studied. Rats were allocated to each group by matching by body weight and systolic blood pressure. Oxonic acid, potassium salt was dosed daily by gavage using flexible polyethylene tube and a syringe in morning hours (750 mg/kg BW, Sigma-Aldrich Chemical, St Louis MO, USA) for a total of 5 weeks, including weekends.

Diets: Rat Chow AIN-93 (Purified Diets for laboratory rodents) was purchased from Dyets Inc. Bethlehem PA. Two probiotic containing formulas were prepared and stored at -20C then shipped to Mexico. The compositions of the formulas were as below:

Placebo–Cream of wheat

Formula 1- L acidophilus KB27 (5.0 B CFU/day), L rhamnosus KB79(5.0 B CFU/day), Xylooligosaccharide-50.0 mgs per day

Formula 2- L acidophilus KB27 (5.0 B CFU/day), L rhamnosus KB79(5.0 B CFU/day), Xylooligosaccharide-50.0 mgs per day, curcumin-25.0 mgs/day

The formulas were mixed into the rat chow and made into 5.0-gram balls.

Both strains of probiotics are GRAS certified and are manufactured in a cGMP facility in the USA. All ingredients are made in the USA. Patent application on the product has been submitted and is pending.

Normal regular diet and diet containing placebo was prepared and stored in the same way. To guarantee a uniform feeding, each rat received two balls of food in the morning, three hours after OA dosing and two balls at night. All animals consumed all food during the day. Tap water was provided *ad libitum* to all groups.

The following groups were included:

C = Control group. Normal healthy rats receiving normal regular diet
HU-ND = Oxonic acid-induced hyperuricemia receiving normal regular diet
HU-P = Oxonic acid-induced hyperuricemia receiving placebo containing diet
HU-F1 = Oxonic acid-induced hyperuricemia receiving probiotics formula 1 containing diet.

HU-F2 = Oxonic acid-induced hyperuricemia receiving probiotics formula 2 containing diet.

Oxonic acid dosing and probiotics feeding were started at the same time point. No adverse events were observed during the 5-week follow-up, and all rats reached the end of the study.

Systolic blood pressure measurements, urine collection, and blood samples were obtained at 3 and 5 weeks, at this latter point fecal samples were also collected. At the end of the study, rats were sacrificed by deep anesthesia with inhaled isoflurane and exsanguination via abdominal aorta with a heparinized syringe. The collected blood was centrifuged, and plasma separated and frozen until further analysis. Immediately, the kidneys were washed by perfusion with cold PBS and right kidney excised separated in cortex and medulla and stored in liquid nitrogen until further processing. The left kidney was fixed by perfusion for histological analysis. Samples of the small intestine (ileum) were also taken and stored in liquid nitrogen.

### Endpoints

The primary endpoint was plasma UA concentration. The secondary endpoints included systolic blood pressure, urine UA, urine oxidative stress (TBARS), creatinine clearance, urine and plasma nitrites and nitrates, urine N-acetyl-beta-D-glucosaminidase (NAG) activity (marker of tubular damage), renal histology,including Periodic acid Schiff´s forinflammation, Masson’s trichrome for fibrosis and immunohistochemistry for alpha-smooth muscle actin (GeneTex, Irvine CA USA) for arteriolopathy, and markers of oxidative stress (protein oxidation and lipid peroxidation) and uric acid concentration in renal cortex homogenates. Also, the expression of the urate transporter ABCG2 (GeneTex, Irvine CA USA) in ileum was assessed by western blot. Microbiome analysis was performed in fecal samples taken at baseline and the end of the study.

### Measurements

Uric acid in plasma and urine were measured using a commercial enzymatic kit (Sekisui, Diagnostics. Charlottetown, PE. Canada). 5-week plasma samples were in addition, tested using a colorimetric assay (Quantichrom Uric Acid Assay, BioAssay Systems. San Francisco CA, USA).

Urine TBARS and products of nitric oxide metabolism in plasma and urine (nitrates and nitrites) were measured with commercial kits (Cayman Chemical, Ann Arbor, Michigan. USA) Plasma and urine creatinine were measured with a commercial kit (SpinReact. Girona, Spain) and creatinine clearance calculated.

Urine NAG activity was determined using 4-nitrophenyl-N-acetyl-beta-D-glucosaminide as substrate. One unit of enzymatic activity (U) represents the amount of enzyme, which hydrolyzes one micromole of substrate per min at 37°C [31]. The results were expressed as U/24 h.

Renal cortex oxidative stress: The determination of carbonyl groups in the proteins of renal cortex homogenates was measured using the reaction with 2,4-Dinitrophenylhydrazine (DNPH) as previously described [32]. For the 4-HNE assay, 50 mg of kidney cortex were homogenized in ice-cold phosphate buffered saline (PBS), the colorimetric assay was performed as previously described (27). The results were expressed as nmol of 4-HNE/mg protein.

Renal cortex uric acid content: Uric acid was extracted from the renal cortex accordingly to methods previously published. Uric acid concentration was measured with a commercial enzymatic kit (Sekisui, Diagnostics. Charlottetown, PE. Canada) and corrected by protein concentration (Bradford method)

### Microbiome analysis

DNA was extracted from fecal samples using the QIAamp PowerFecal Kit (QIAGEN, Carlsbad, CA). Bacterial profiles were determined by broad-range amplification and sequence analysis of 16S rRNA genes following our previously described methods [33–36]. In brief, amplicons were generated using primers that target approximately 400 base pairs of the V3V4 variable region of the 16S rRNA gene. PCR products were normalized using a SequalPrep^TM^ kit (Invitrogen, Carlsbad, CA), pooled, lyophilized, purified and concentrated using a DNA Clean and Concentrator Kit (Zymo, Irvine, CA). The denatured PCR amplicon pool was diluted to 15 pM and spiked with 25% of the Illumina PhiX control DNA prior to loading the sequencer. Illumina paired-end sequencing was performed on the Miseq platform using a 600 cycle version 3 reagent kit.

Illumina Miseq paired-end reads were aligned to rat reference genome rn4 with bowtie2 and matching sequences discarded [37]. As previously described, the remaining non-rodent paired-end sequences were sorted by sample via barcodes in the paired reads with a python script [38]. Sorted paired-end sequence data were deposited in the NCBI Sequence Read Archive under project accession number PRJNA449940. The sorted paired reads were assembled using phrap [39, 40]. Pairs that did not assemble were discarded. Assembled sequence ends were trimmed over a moving window of 5 nucleotides until average quality met or exceeded 20. Trimmed sequences with more than 1 ambiguity or shorter than 350 nt were discarded. Potential chimeras identified with Uchime (usearch6.0.203_i86linux32) [41] using the Schloss [42] Silva reference sequences were removed from subsequent analyses. Assembled sequences were aligned and classified with SINA (1.3.0-r23838) [43] using the 418,497 bacterial sequences in Silva 115NR99 [44] as reference configured to yield the Silva taxonomy. Operational taxonomic units (OTUs) were produced by clustering sequences with identical taxonomic assignments. This process generated 6,840,738 sequences for 60 samples (median sample size: 116,081; interquartile range: 105,788–121,149). The median Goods coverage score was ≥ 99.96% at the rarefaction point of 54,698 sequences.

### Statistical analysis

Values were expressed as the mean ± standard deviation (SD). Differences between groups were evaluated by two-way ANOVA or one-way-ANOVA with Tukey’s correction for multiple comparisons. The R and Explicet [45] software packages were used for all microbiome analyses and figure generation. Differences in overall community composition between treatment groups were assessed using the permutation-based multiple analysis of variance (PERMANOVA) test of Bray-Curtis dissimilarities, as implemented in the R vegan package.[46] P-values were obtained through 1,000,000 permutations. Individual OTUs that differed in relative abundance or prevalence between groups were identified using a non-parametric Kruskal-Wallis test. Measures of alpha-diversity (Chao1, evenness, Shannon complexity) were estimated using Explicet (v2.10.5,) [45] through 1,000 replicate re-samplings at the rarefaction point of 54,698 sequences; between-group differences in these indices were assessed by ANOVA.

## Results

All animals started with similar body weights (313±2 g) and were fed 20 g of diet each day. All groups had similar linear growth trends over the 5-week experiment (C: Slope = 1.73±0.2, r = 0.51; HU+ND: Slope = 1.79±0.13, r = 0.68; HU+P: Slope = 1.54±0.17, r = 0.54; HU+F1: Slope = 1.36±0.15, r = 0.54; HU+F2: Slope = 1.20±0.17, r = 0.42). Rats on the HU+F2 diet tended to gain less body weight, but this was not significant.

### Primary endpoint

#### Probiotics supplements prevented hyperuricemia in oxonic acid-dosed rats

**Fig 1:**
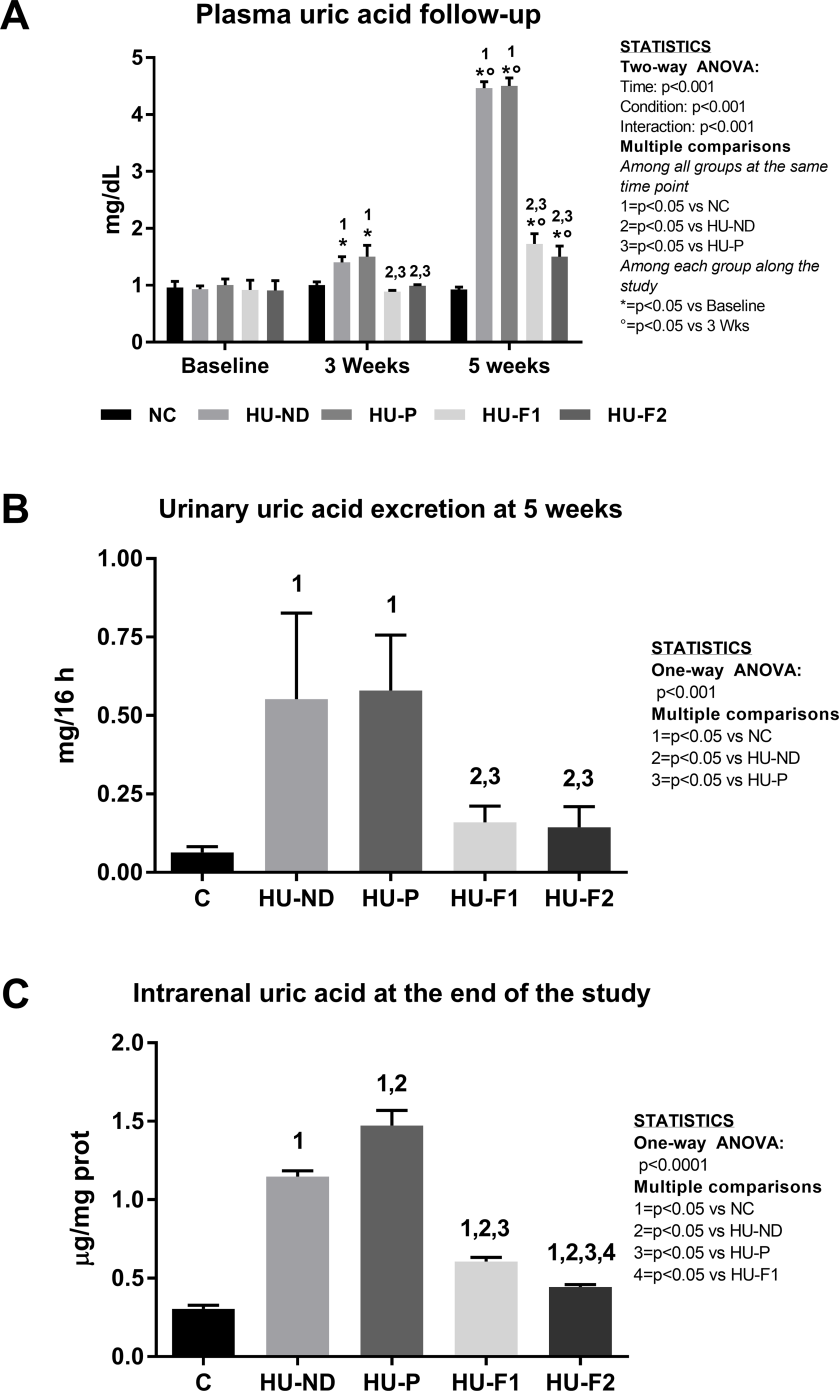
Effect of probiotics-containing diets on uric acid blood levels, urine excretion, and renal tissue concentration. A) OA administration induced the elevation of UA in plasma, and this effect was more evident after 5weeks. Both probiotics formulas given in diet successfully prevented the rise of plasma UA. B) Urine excretion of UA was increased in OA-induced hyperuricemic rats, receiving normal and placebo diets. Both probiotics formulas prevented the increased UA urine excretion. C) Renal UA concentration was also significantly increased in hyperuricemic groups, and this effect was also prevented by probiotics-containing diets. N = 6 each group.

At baseline, all groups had comparable values of PUA (Fig 1A). In the control (C) group, PUA remained unchanged during the 5 weeks of follow-up. OA administration induced a mild but significant increase in PUA in rats receiving normal diet and placebo after 3 weeks, and this effect was further accentuated at 5 weeks of the follow-up. The co-administration of F1 and F2 in rats receiving OA prevented the rise of PUA at 3 weeks and 5 weeks in a comparable manner. Although at the end of the study HU-F1 and HU-F2 groups had higher PUA concentrations compared to their respective baselines, still the levels were significantly lower compared to HU-ND and HU-P groups at 3 weeks with even greater differences in plasma uric acid at 5 weeks of follow-up. These findings were also confirmed with an improved colorimetric method that measures uric acid using 2,4,6-tripyridyl-s-triazine that forms a blue colored complex specifically with iron in the presence of uric acid (data not shown). This method provided comparable values of plasma uric acid than the enzymatic one in the different groups at 5 weeks, as it was shown in the correlation analysis between the values of both methods (y = 1.06X-0.14, r = 0.99, p<0.0001).

Urinary excretion of uric acid (Fig 1B): At the end of the study, there was a significant increment in the urine excretion of UA in the oxonic acid-dosed groups that received normal diet and placebo compared to normal control rats. F1 and F2 prevented the rise in uric acid urinary excretion resulting from oxonic acid-induced hyperuricemia.

Intrarenal uric acid (Fig 1C): Oxonic acid administration induced the accumulation of intrarenal uric acid in the oxonic acid-dosed groups receiving normal diet and placebo and this effect was better observed in the HU-P group. F1 and F2 feeding partially but significantly prevented the uric acid accumulation in renal tissue.

### Secondary endpoints

#### Probiotics supplementation prevented renal alterations induced by hyperuricemia

Systolic blood pressure (Table 1): There were no changes in SBP among the groups at Baseline. At weeks 3 and 5, Normal and placebo diet rats administered oxonic acid had a mild increase in SBP compared to normal controls (NC) that only reached statistical significance in the normal diet hyperuricemic group. At 5 weeks, F1 and F2 prevented the increment of SBP induced by oxonic acid in the rats receiving the normal and placebo diets. In those groups (F1 and F2), the final SBP tended to be lower in comparison to baseline in the same groups, although it was not significant.

**Table 1:** Systolic blood pressure follow-up. N = 6 each group.

	Control	HU+ND	HU+P	HU+F1	HU+F2
**Baseline**	124±11	128±5	119±11	128±4	123±10
**3 weeks**	119±9	136±10^**1**^	133±3	124±9	125±5
**5 weeks**	126±7	135±7	138±4*	118±8^**2,3**^	121±6^**3**^

Statistics:

Two-way ANOVA Time = ns; Condition p<0.001; Interaction p<0.01

Multiple comparisons

Among all groups at the same time point: 1 = p<0.05 vs C; 2 = p<0.05 vs HU-ND; 3 = p<0.05 vs HU+P.

Among each group along the study

* = p<0.05 vs Baseline

**Fig 2:**
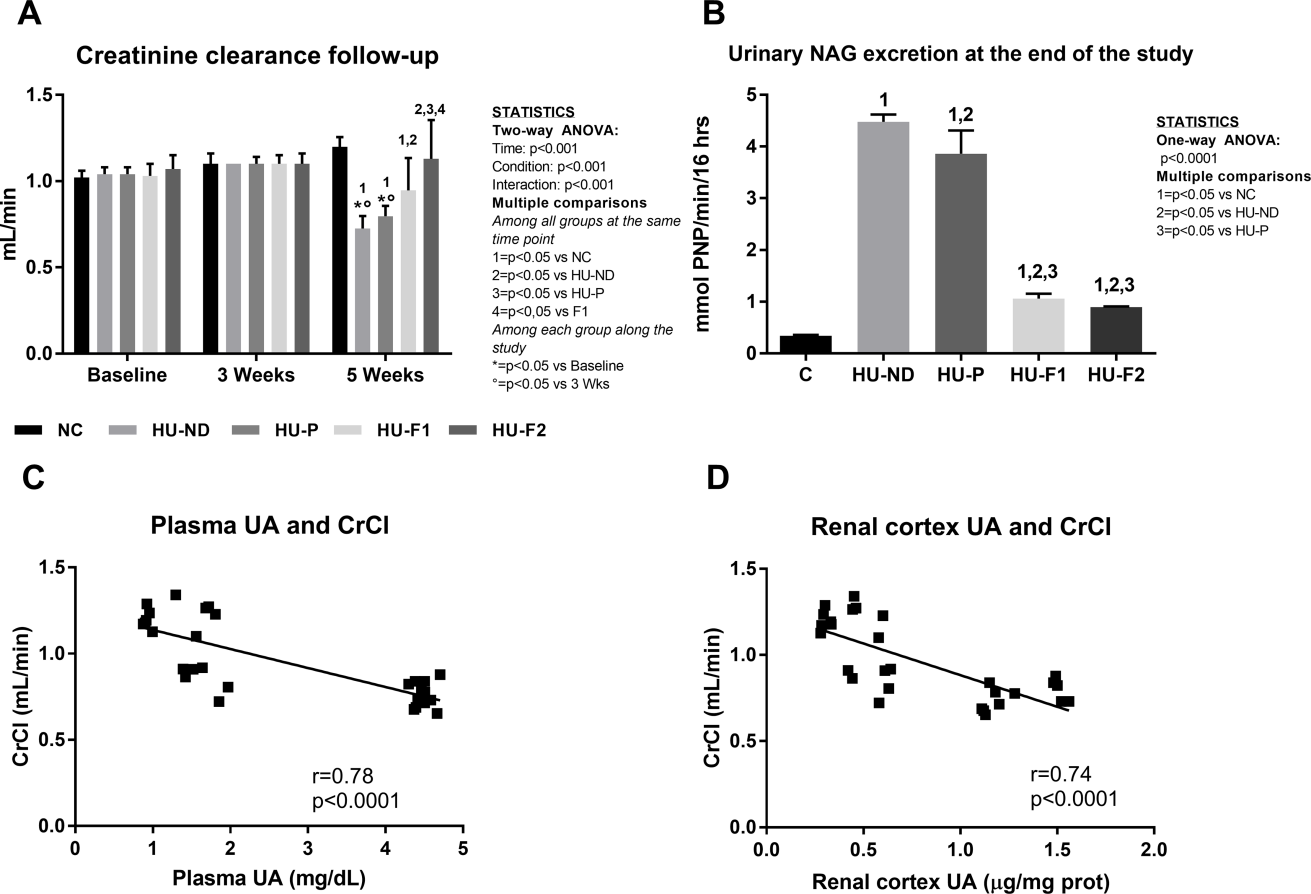
Effect of probiotics-containing diets on renal changes induced by hyperuricemia. A) Creatinine clearance was significantly reduced in both groups of hyperuricemic rats at 5 weeks. This effect was partially prevented by probiotics-containing diets and better observed with Formula 2. B) The marker of tubular damage NAG was increased by hyperuricemia. Both probiotics diets prevented this effect. N = 6 each group. C) Correlation between plasma UA concentration and CrCl. D) Correlation between renal cortex UA concentration and CrCl.

Creatinine clearance (Fig 2A): There were no changes in the CrCl among the groups at Baseline and after 3 weeks of follow-up. At 5 weeks, normal diet and placebo diets had a significant decrement in CrCl (-30 to -40%). F1 partially but significantly prevented this effect. Moreover, F2 had a significantly better effect compared to F1 and fully prevented the fall in CrCl induced by HU. We also found negative correlations between CrCl and final plasma uric acid (r = -78, p<0.0001, Fig 2C), and CrCl and renal cortex uric acid (r = -0.74, p<0.0001, Fig 2D).

Urinary N- acetyl-beta-D-glucosaminidase (NAG) activity (Fig 2B): NAG urinary activity is a marker of tubular injury. Oxonic acid-induced hyperuricemia produced an eight-fold increase in the urinary activity of this enzyme in groups that received normal diet or placebo. Feeding with F1 and F2 partially prevented the rise of NAG activity at the end of the study.

**Fig 3:**
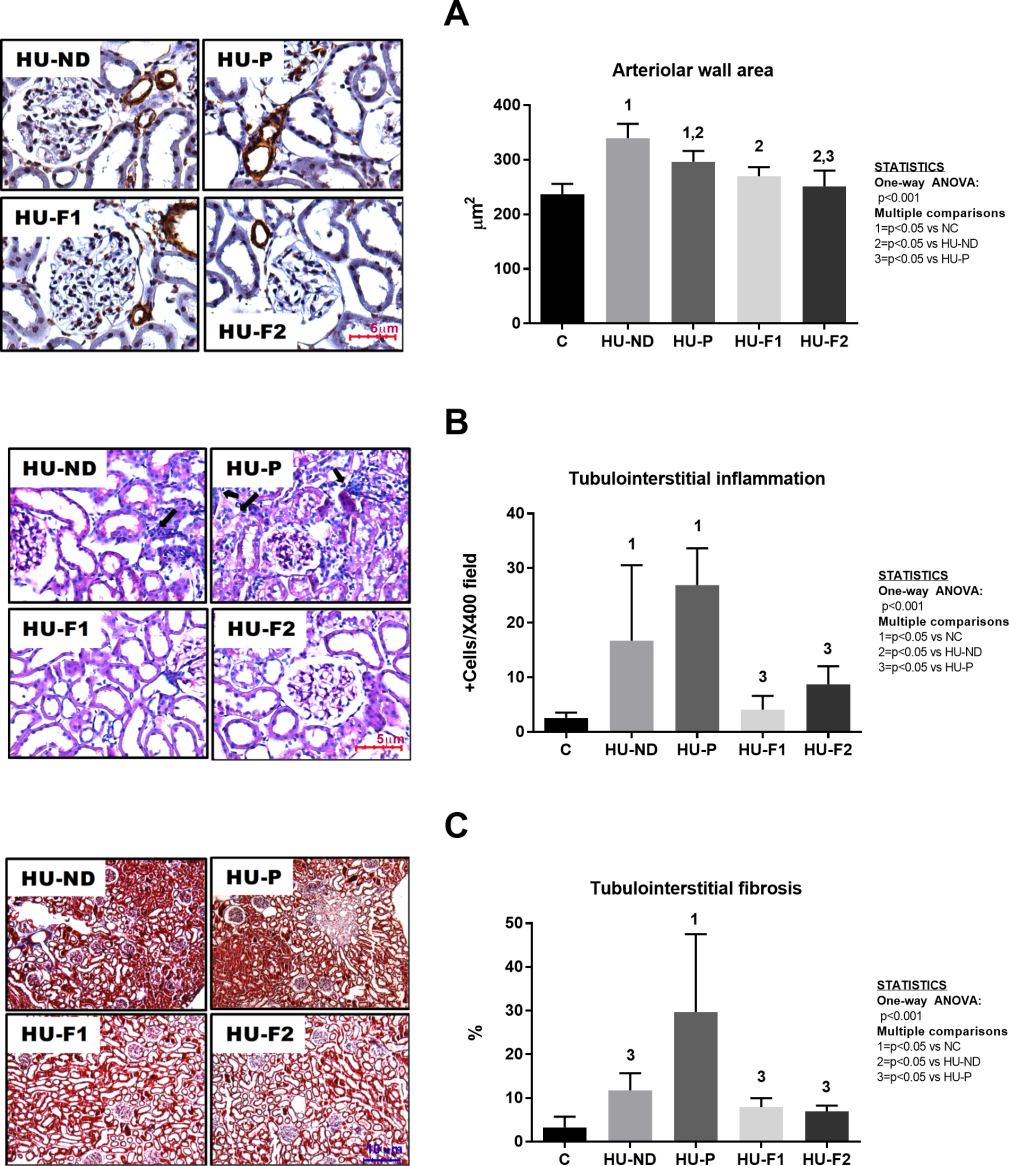
Effect of probiotics-containing diets on renal structural changes induced by hyperuricemia. A) Arteriolar wall area of renal vessels (immunohistochemistry for vascular smooth muscle alpha-actin, 400X) was increased in hyperuricemic groups. Probiotics intake prevented this alteration; this effect was greater observed in the group that received Formula 2. B) Tubulointerstitial inflammation was evaluated with Periodic acid Schiff´s staining, 400X. Hyperuricemia induced inflammatory infiltration that was prevented by probiotics supplementation in the diet. C) Tubulointerstitial fibrosis was evaluated with Masson´s trichromic staining, 100X. Hyperuricemia increased tubulointerstitial collagen deposition; the effect was prevented by probiotics intake. N = 6 each group.

Histopathological findings (Fig 3): Renal histologic analysis performed at the end of the study (after 5 weeks of oxonic acid exposure) documented an afferent arteriolopathy in oxonic acid-treated rats with hyperuricemia (defined as an increase in arteriolar wall area, Fig 3A), as well as tubulointerstitial inflammation (Fig 3B) and fibrosis (Fig 3C). These three alterations reached statistical significance in the group that received placebo, while in the normal diet hyperuricemic group, the fibrosis did not reach statistical significance. F1 and F2 feeding prevented those alterations, and the effect was greater observed when compared versus the HU-P group.

#### Probiotics supplementation prevented oxidative stress induced by hyperuricemia

**Fig 4:**
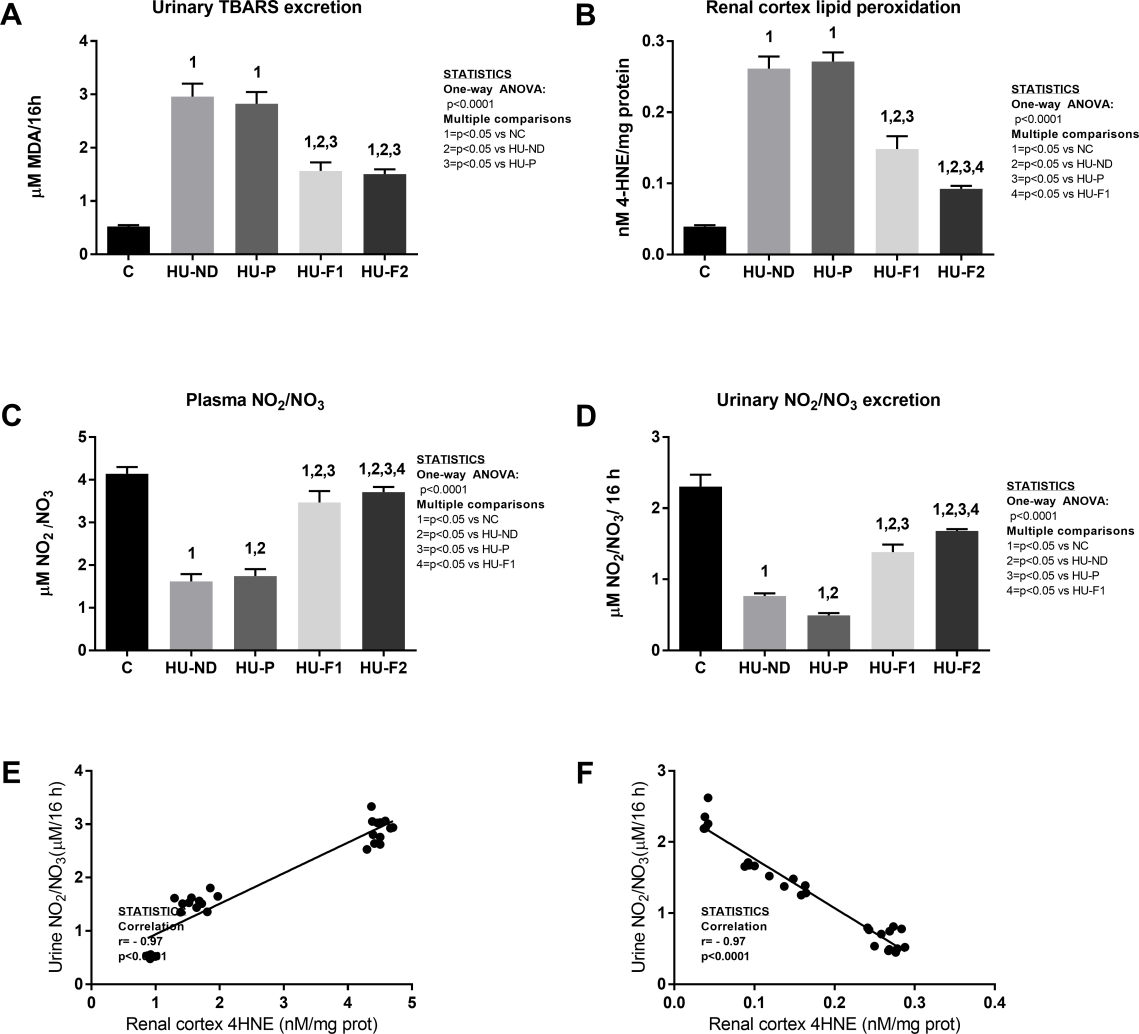
Probiotics supplementation prevented oxidative stress and endothelial dysfunction induced by hyperuricemia. Hyperuricemia induced systemic (A) and renal tissue (B) oxidative stress as well as reduction of NO_2_/NO_3_ byproducts (C,D). Probiotics supplementation prevented both effects. Markers of oxidative stress showed a negative correlation with NO_2_/NO_3_ byproducts (E,F).

**Fig 5:**
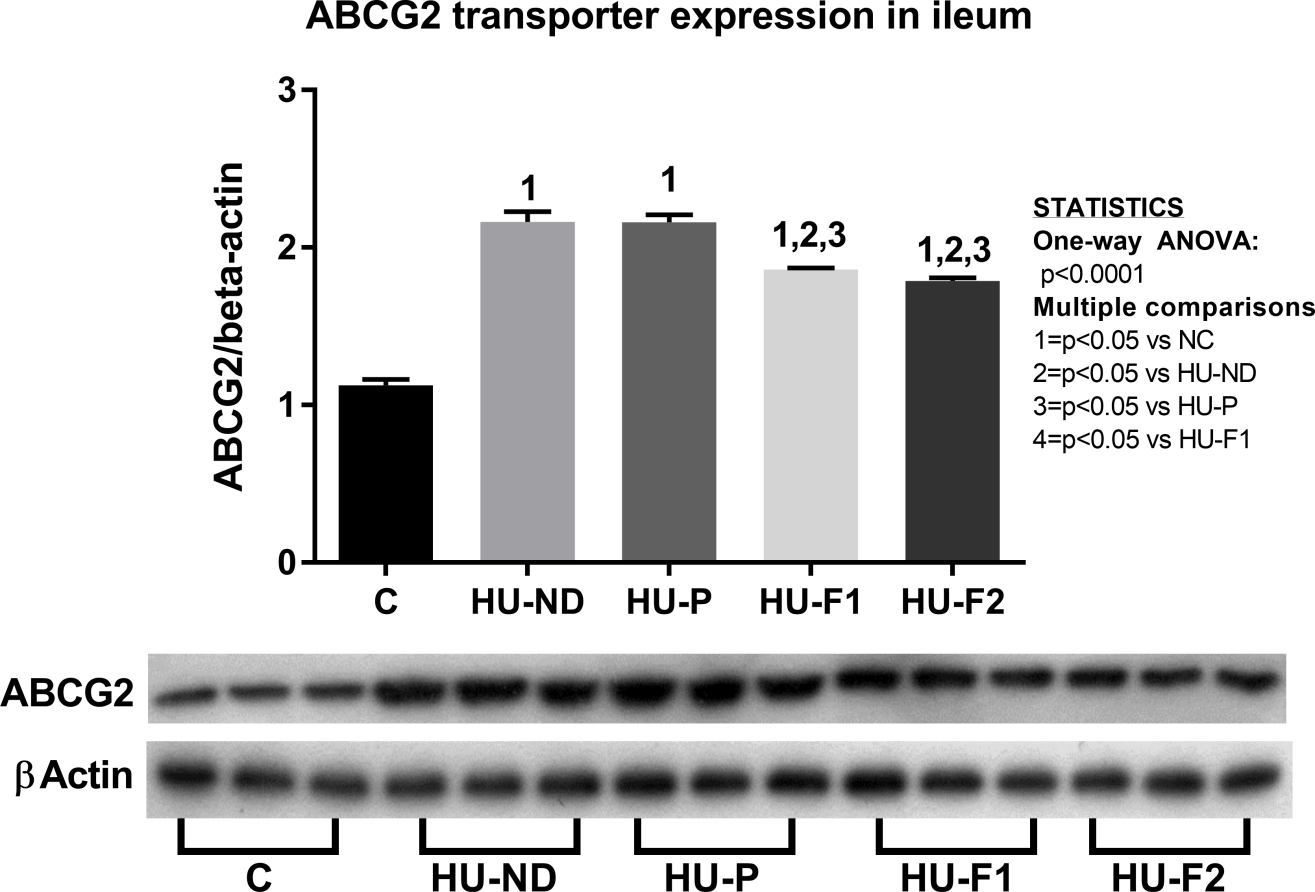
Probiotics supplementation resulted in decreased expression of ABCG2 transporter in the ileum. Hyperuricemia upregulated the expression of ABCG2 by western-blot. Probiotics supplementation prevented ABCG2 overexpression. N = 6 each group.

Systemic oxidative stress (Fig 4A). Thiobarbituric acid reactive species (TBARS) in urine (Fig 5): TBARS urinary excretion is a measure of systemic lipid peroxidation secondary to oxidative stress. Hyperuricemic groups receiving normal diet or placebo had a 5-time increment in the excretion of urinary TBARS. The feeding with F1 and F2 partially but significantly ameliorated that effect.

Renal cortex oxidative stress (Fig 4B): At the end of the study oxonic acid-induced hyperuricemia significantly increased lipid peroxidation in the renal cortex. This effect was partially prevented by F1, while F2 had a significantly better effect in comparison to F1.

Plasma concentration and urinary excretion of nitric oxide metabolism byproducts (NO_2_/NO_3_) (Fig 4C and 4D): Hyperuricemia induced a marked reduction in plasma and urinary NO_2_/NO_3_ at the end of the study. F1 and F2 feeding partially rescued nitric oxide byproducts plasma and urinary levels, and this effect was better observed in F2 fed rats.

Correlation between markers of oxidative stress and NO_2_/NO_3_ byproducts (Fig 4E and 4F): We also found strong correlations between systemic (Fig 4E) and renal (Fig 4F) markers of oxidative stress and NO_2_/NO_3_ byproducts.

#### Probiotics supplementation resulted in decreased expression of ABCG2 transporter in the ileum

Oxonic acid-induced hyperuricemia significantly increased the expression of this transporter. Probiotics supplements partially reduced this overexpression (Fig 5).

#### Microbiome analysis

**Fig 6:**
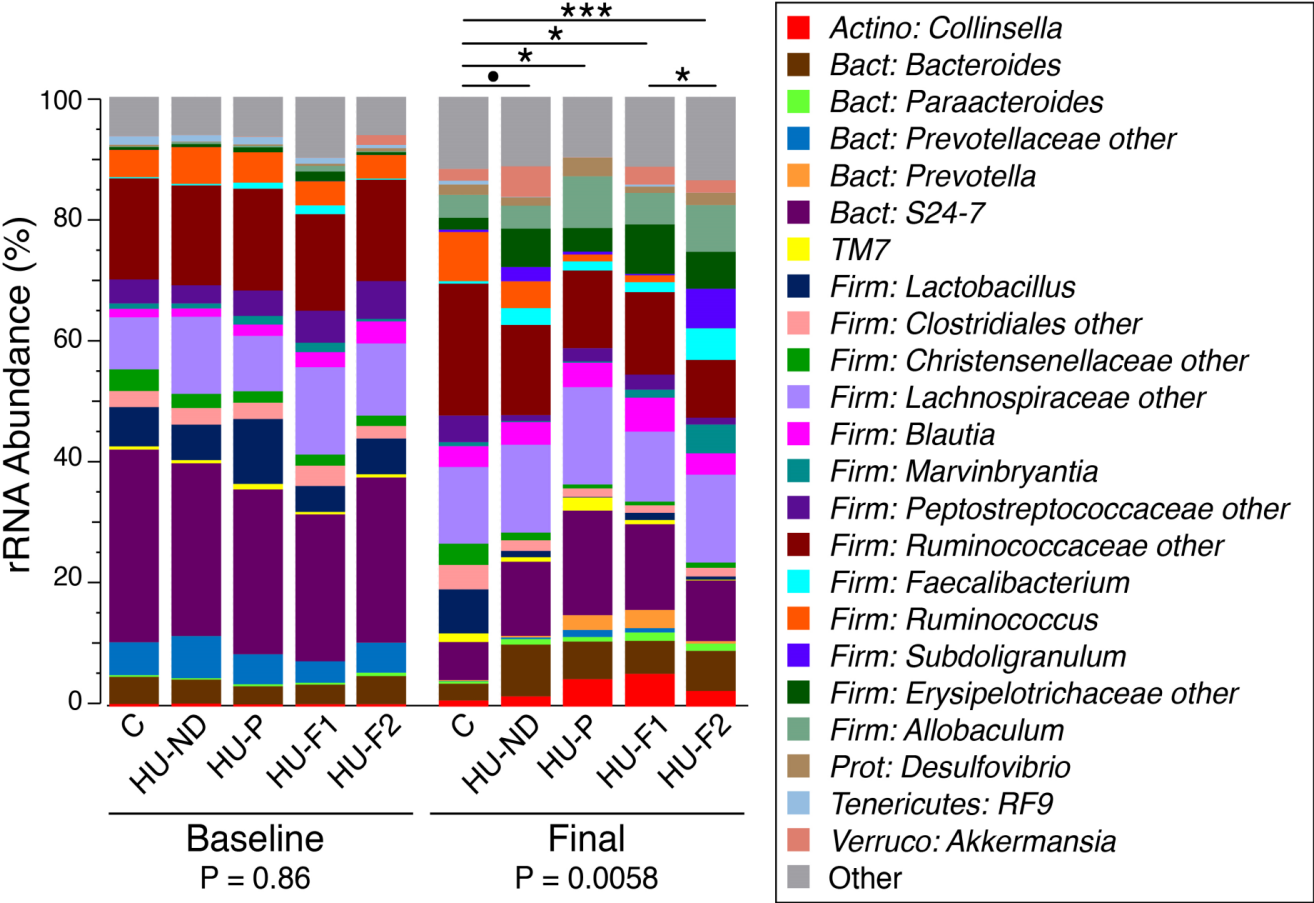
Distributions of bacterial taxa differ upon OA and probiotic treatment. Bar charts summarize the relative abundances of bacterial genera averaged within each treatment group and at Baseline and the 5-week time point (Final). Overall differences in microbiota across all five treatment groups are indicated by PERMANOVA p-values underneath each time point. PERMANOVA p-values for pairwise comparisons of treatment groups are indicated above the bar charts (•: p<0.1; *: p<0.05; **: p<0.01; ***:p<0.001). For simplicity of presentation, all taxa with relative abundance <0.05% were aggregated into the “Other” category. Treatment group labels are defined in the text.

16S rRNA gene-based profiling of fecal specimens collected at Baseline and week 5 was successful for all samples; all sequence libraries had Good’s coverage indices >99%, indicating sufficient depth of sequence coverage was achieved for each sample. At Baseline, no differences in overall bacterial community composition were observed between treatment groups (PERMANOVA p = 0.86; Fig 6). In contrast, at the 5-week time-point, significant differences were observed in fecal microbiota both across all treatment groups (PERMANOVA p = 0.0058) and in pairwise comparisons between the untreated animals (group C) and each of the OA-treated groups (Fig 6). In particular, differences in the microbiota of group C (OA naïve) and HU-ND (OA treated) approached significance (PERMANOVA p = 0.067).

**Fig 7:**
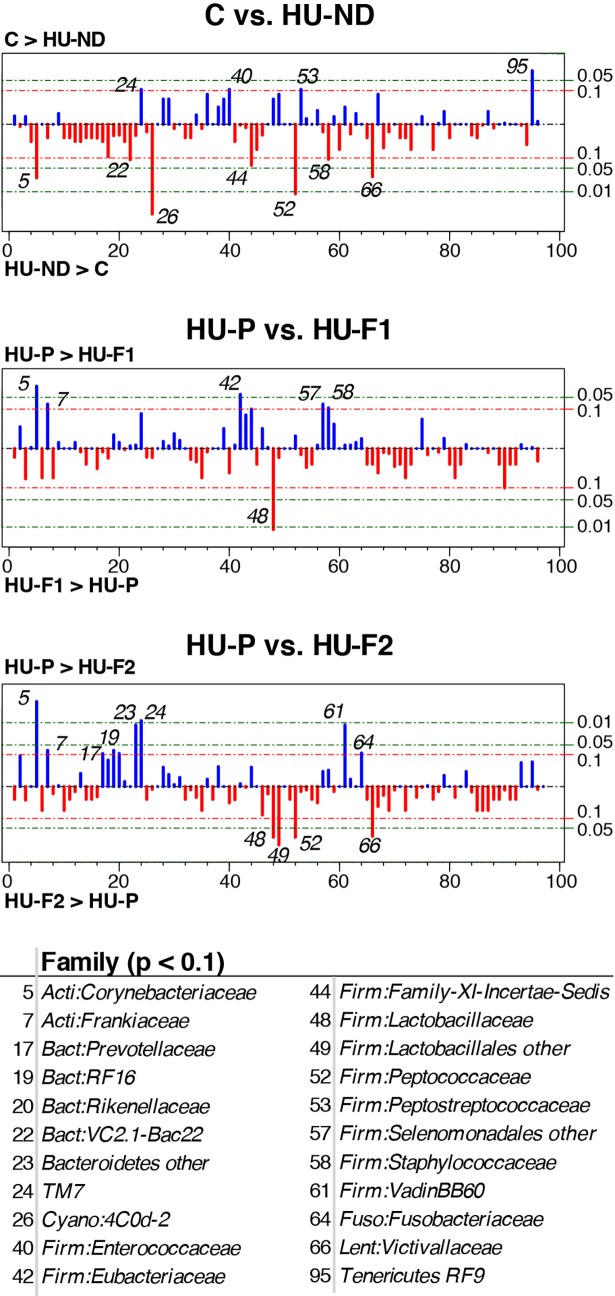
Individual taxa differing between selected treatment groups. Bacterial families are enumerated along the x-axes, while y-axes display the–log(p-values) for each Kruskal-Wallis statistical test, which results in longer lines for smaller p-values.Blue lines extending upwards indicate taxa that were more abundant in the first comparison group listed at the top of each plot, while red lines extending downwards indicate the opposite. Taxa with p-values less than 0.1 in any test are listed below the plots. Family names are preceded by phylum names: Acti = Actinobacteria, Bact = Bacteroidetes, Cyano = Cyanobacterium, Firm = Firmicutes, Fuso = Fusobacteria, Lenti = Lentisphaerae, Tene = Tenericutes. Treatment group labels are defined in the text.

Several bacterial families differed in relative abundance between these two treatment groups (Fig 7), including *Corynbacteriaceae* (p = 0.026), Cyanobacterium 4C0d-2 (p = 0.0022), *Peptococcaceae* (p = 0.0087), *Victivallaceae* (p = 0.028), and Tenericutes RF9 (p = 0.026).

Of note, neither HU-F1 nor HU-F2 probiotic treatments restored the microbiota to that of the OA-naïve group C, as indicated by the significant differences observed between both probiotic-treatment groups and group C (PERMANOVA p = 0.01 and p<0.001, respectively). In contrast, no significant differences were evident when comparing overall microbiota compositions of either HU-F1 or HU-F2 probiotic treatment groups with the placebo group (HU-P, PERMANOVA p = 0.89 and p = 0.12, respectively). *Lactobacillaceae* were, however, significantly enriched in both HU-F1 (p = 0.0087) and HU-F2 (p = 0.026) compared to HU-P, whereas two families of Actinobacteria (*Corynebacteriaceae* and *Frankiaceae*) were concomitantly reduced in both HU-F1 (p = 0.026 and p = 0.074, respectively) and HU-F2 (p = 0.0022 and p = 0.074, respectively). Finally, significant microbiota differences were observed between HU-F1 and HU-F2 (p = 0.037) suggesting an effect of curcumin treatment on bacterial community composition; however, *Lactobacillaceae* abundances did not differ between these two groups (p = 0.70).

### Discussion

Hyperuricemia is common in the general population and has been associated with the presence of hypertension, acute and chronic renal injury and metabolic alterations such as diabetes and fatty liver. Experimental evidence suggests that elevated uric acid might not be simply secondary to these conditions, but might drive them by causing intracellular oxidative stress, endothelial dysfunction, mitochondrial and endoplasmic reticulum alterations that lead to systemic and renal vasculopathy, vasoconstriction, inflammation, and fibrosis.

While clinical proof that hyperuricemia has a causal role in these conditions is needed, another concern is that current treatments for hyperuricemia can be associated with significant side effects. Therefore, the use of innocuous means to reduce serum uric acid could be clinically important. In this regard, probiotic supplementation could represent an approach to treat chronic diseases without causing undesirable side effects. Given that gut microbiota already are known to modulate plasma uric acid levels (13), we attempted to boost the microbial capacity by administering uricolytic bacteria as a probiotic.

In the present study, we tested the feasibility of two probiotic preparations to lower plasma uric acid using the model of mild hyperuricemia induced by oxonic acid. One of the probiotics also contained curcurmin, which has beneficial properties as an antioxidant and anti-inflammatory agent [47]. We also tested whether these supplements provide therapeutic benefit in the renal alterations induced by hyperuricemia. Both preparations were well tolerated by the rats as they had a similar growth curve during the 5 weeks of the follow-up.

#### Primary outcome: Uric acid concentrations in plasma, urine and renal cortex

We observed a gradual increment in plasma UA following administration of oxonic acid in rats receiving normal and placebo diets. The groups on those diets finished with a significant ~3-fold increment of plasma UA compared to week 3. We previously reported that oxonic acid dosing in rats induced 1.5–2 time increase in plasma uric acid levels after 5 weeks of treatment from baseline (from 1 mg/dL to 1.5–2 mg/dl) [48, 49]. In the present study, we observed a higher increase in this parameter after 5 weeks (4.5 fold). We do not have a definite explanation for this, but it might be related to the components of the diet in the present study. Despite a higher increase in plasma UA induced by normal and placebo diets, both probiotics formulations, F1 and F2, prevented increased UA despite rats receiving oxonic acid, and this effect was better observed at 5 weeks. Uric acid excretion was also increased in hyperuricemic rats receiving normal and placebo diets, but also was prevented by both probiotics supplements, suggesting that a higher proportion of uric acid was eliminated via intestinal uricolysis. Finally, intrarenal uric acid concentrations were significantly increased in hyperuricemic rats, an effect that was prevented by probiotics supplementation. Interestingly, F2, the supplement containing curcumin, provided a greater benefit on this parameter. On the other hand, it is conceivable that probiotics metabolize oxonic acid thus preventing uricase blockade. However, this seems unlikely since oxonic acid is rapidly absorbed into the portal vein where it is taken up in the liver and inhibits liver uricase. Additional models of hyperuricemia, not involving oxonic acid, would help to confirm our findings.

#### Secondary outcomes: Renal changes and hypertension

The oxonic-acid model of mild hyperuricemia is reproducible in rats and mice and consistently elevates systolic blood pressure, increases oxidative stress and causes renal alterations characterized by vasoconstriction, arteriolopathy, endothelial dysfunction and tubulointerstitial inflammatory and fibrotic changes; those effects were observed without the precipitation of uric acid crystals (31–33). This model has been useful to show the deleterious effects of uric acid; thus, several epidemiological studies have found that hyperuricemia has a predictive value for the development of hypertension and intense clinical research is ongoing to clarify the role of uric acid on renal and metabolic diseases [50].

As previously reported, oxonic acid-induced hyperuricemia produced a mild increment in systolic blood pressure. Treatments that reduce UA levels, prevent oxidative stress or preserve nitric oxide bioavailability can prevent this effect [48, 49, 51, 52]. In the present study, prevention of hyperuricemia by feeding rats with two different formulations of probiotics also had a beneficial effect on blood pressure. However, because the number of animals tested was limited these results should be interpreted with caution.

Asymptomatic hyperuricemia can cause mild renal damage after 5 weeks in rats [53, 54]. In the present study, we found a significant decrement in creatinine clearance and an increment in NAG urinary excretion along with mild structural damage including increased wall arteriolar area and tubulointerstitial inflammation and fibrosis in both hyperuricemic groups. Feeding with F1 and F2 supplements prevented such renal alterations, and the beneficial effects were enhanced when curcumin was included in the formula. Curcumin has independently been reported to provide therapeutic benefit in acute and chronic renal injury by means of its antioxidant and anti-inflammatory capacity through the activation of the Keap1-Nrf2 pathway [47]. Curcumin has also been proposed to have prebiotic potential [55]. Therefore, it is possible that a combination of both probiotic bacteria that can lower uric acid and curcumin could explain the enhanced benefit provided by F2. In this regard, both formulations showed similar potency for preventing hyperuricemia. However, the formula containing curcumin (F2) further reduced UA concentration in renal cortex tissue and this correlated with an improved CrCl and less intrarenal lipid peroxidation.

Intriguingly, we had expected a higher increase in SBP as UA plasma concentration reached a 4.5-fold increase in comparison to previous studies where the rise was of 2-3-fold. We do not have an explanation for why this was not observed. However, creatinine clearance, a surrogate marker of glomerular filtration rate decreased by 30–40%, a higher value than previously reported [49], suggesting profound renal vasoconstriction induced by the higher levels of uric acid.

Some evidence suggests that a primary mechanism for uric acid to induce damage is through increase oxidative stress and reduced NO bioavailability. Therefore we measured the urinary excretion of thiobarbituric acid reactive substances, in renal cortex homogenates 4-hydroxynonenal, and in plasma and urine the concentration of nitrates and nitrates as markers of oxidative stress and NO bioavailability. We confirmed that prevention of hyperuricemia, accomplished in this study by probiotics supplementation, also prevented the increase in systemic and intrarenal oxidative stress and reduced NO biovailability. Moreover, we found negative and significant correlations between lipid peroxidation markers and nitric oxide metabolites, suggesting that oxidative stress decreased the bioavailability of nitric oxide. This finding would also suggest that the reduction in creatinine clearance probably resulted from renal vasoconstriction, mediated by oxidative stress and reduced nitric oxide bioavailability. Importantly, the prevention of hyperuricemia aimed at the feeding of F1 and F2 provided benefit by preserving renal function, and the F2 probiotic containing curcumin better accomplished this.

ABCG2 transports uric acid from systemic circulation into the small intestine. It is expressed in various segments of the small and large intestine. In 5/6 nephrectomy rats, the expression of this transporter is increased, mainly in the ileum [12, 56] which may represent compensation to maintain serum uric acid levels in patients with chronic kidney disease in which urinary excretion is relatively impaired [57]. Therefore, we evaluated ABCG2 expression in the ileal segment and found that hyperuricemia induced a 200% increase in its expression, while F1 and F2 treatments prevented this effect. As previously reported, oxonic acid-induced hyperuricemia increased ABCG2 transporter expression in the ileum [58]. The fact that F1 (+1.7 times) and F2 (+1.6 times) partially, but significantly, prevented this effect might be related to a better control of uric acid levels by the probiotics such that upregulation of ABCG2 was lower, but significantly higher compared to normal control group. Despite these findings, we cannot rule out a compensatory increase in ABCG2 activity as urine excretion and renal tissue concentrations were decreased by F1 and F2. Another possible effect is that normalization of UA levels with probiotics facilitated the localization of ABCG2 transporter on the plasma membrane as hyperuricemia downregulates the cell surface expression of this transporter [59].

OA treatment was associated with modest changes in the overall composition of the fecal microbiota, along with significantly altered relative abundances of multiple bacterial taxa belonging to a wide range of phyla (e.g., Actinobacteria, Bacteroidetes, Firmicutes, Lentisphaerae, Tenericutes, TM7). However, the benefits of probiotic treatment to reverse OA-induced pathology were not paralleled by a restoration of the fecal microbiota, at least over the limited time-course of this experiment. Therefore, OA-induced dysbiosis may be immaterial to the disease process in this model system. Alternatively, the relatively small number of animals included in these studies likely reduced statistical power to detect all but the largest effect sizes; larger treatment groups coupled with more prolonged courses of OA and probiotic exposures might be necessary to determine the role of the gut microbiota in hyperuricemia more conclusively.

In summary, we demonstrate for the first time the ability of probiotics containing uricolytic bacteria to lower serum uric acid in hyperuricemic animals with beneficial consequences on blood pressure and renal disease. We suggest clinical studies are needed to evaluate this approach for treating hyperuricemia in humans.

## Supporting information

S1 File Individual data for the provided graphs can be found at “Dataset PlosOne.xlsx” file. (XLSX)
